# A luminescence-based reporter to study tau secretion reveals overlapping mechanisms for the release of healthy and pathological tau

**DOI:** 10.3389/fnins.2023.1196007

**Published:** 2023-06-05

**Authors:** Dianne Marquez Lopez, Connor J. Maltby, Hannah Warming, Nullin Divecha, Mariana Vargas-Caballero, Mark J. Coldwell, Katrin Deinhardt

**Affiliations:** School of Biological Sciences, University of Southampton, Southampton, United Kingdom

**Keywords:** tauopathy, tau secretion, split luciferase reporter, neuronal activity, biosensor

## Abstract

In Alzheimer’s disease, tau pathology is thought to spread via a prion-like manner along connected neuronal networks. For this to occur, the usually cytosolic tau protein must be secreted via an unconventional mechanism prior to uptake into the connected neuron. While secretion of healthy and pathological tau has been documented, it remains under-investigated whether this occurs via overlapping or distinct processes. Here, we established a sensitive bioluminescence-based assay to assess mechanisms underlying the secretion of pseudohyperphosphorylated and wild-type tau in cultured murine hippocampal neurons. We found that under basal conditions, both wild-type and mutant tau are secreted, with mutant tau being more robustly secreted. Pharmacological stimulation of neuronal activity led to a modest increase of wild-type and mutant tau secretion, whereas inhibition of activity had no effect. Interestingly, inhibition of heparin sulfate proteoglycan (HSPG) biosynthesis drastically decreased secretion of both wild-type and mutant tau without affecting cell viability. This shows that native and pathological tau share release mechanisms; both activity-dependent and non-activity-dependent secretion of tau is facilitated by HSPGs.

## Introduction

Alzheimer’s disease (AD) is a neurodegenerative disease that is characterized by the deposition of amyloid plaques as well as neurofibrillary tangles (NFT) composed of hyperphosphorylated tau. It is well established that the progression of tau pathology in the brain follows a stereotypic anatomical pattern of spread (Braak and Braak, 1991). Evidence from post mortem studies and disease models suggests that pathogenic tau can spread across living and intact neurons in the early stages of the disease, even before overt tangle formation and neurodegeneration (De Calignon et al., 2012; Harris et al., 2012; Liu et al., 2012; Hallinan et al., 2019; Bengoa-Vergniory et al., 2021) likely via trans-synaptic routes (Calafate et al., 2015; Pickett et al., 2017; DeVos et al., 2018), contributing to disease progression.

*In vitro* and *in vivo* studies have demonstrated that misfolded and pathogenic tau is released from an affected cell and taken up by connected cells via synaptic and non-synaptic routes, where it can then template its misfolded conformation onto native cytosolic tau, ultimately initiating a cascade of events leading to the formation of larger tau aggregates and neuronal dysfunction, followed by cell death (Frost et al., 2009; Wu et al., 2013; Pickett et al., 2017; Wang et al., 2017; Hallinan et al., 2019). Native and pathological tau have been detected in conditioned media from cell lines overexpressing tau (Chai et al., 2012), primary neurons (Karch et al., 2012; Pooler et al., 2013; Bright et al., 2015), interstitial fluid and cerebrospinal fluid of mice (Yamada et al., 2011, 2014) and humans (Magnoni et al., 2012), suggesting that tau release is a physiological as well as pathological process. The physiological release of tau has been shown to be modulated by neuronal activity in the brains of mice *in vivo* and neuronal cultures *in vitro* (Pooler et al., 2013; Yamada et al., 2014). Neuronal activity also exacerbates the spreading of tau pathology in connected neuronal networks (Wu et al., 2016; Schultz et al., 2018).

Several lines of evidence have shown that cytosolic tau can be released from neurons via unconventional secretory pathways. Two types of extracellular tau are detected: the majority exists as free protein but a proportion is found in extracellular vesicles (Saman et al., 2012; Pooler et al., 2013; Dujardin et al., 2014; Rodriguez et al., 2017). A proposed mechanism for the release of free tau is the direct translocation across the plasma membrane with the help of heparan sulfate proteoglycans (HSPG) (Katsinelos et al., 2018; Merezhko et al., 2018), which have also been shown to play a role in the uptake of pathological tau from the extracellular space (Holmes et al., 2013; Rauch et al., 2018). To address the question whether physiological and pathological tau follow separate or overlapping secretory mechanisms, we designed a luminescence-based reporter system using the split NanoLuc Binary Technology (NanoBiT). This uses the NanoLuc luciferase enzyme, divided into two subunits, LgBiT (17.6 kDa) and a peptide with high binding affinity (HiBiT; 11 amino acids). Structural complementation of the HiBiT and LgBiT reporters re-constitutes the NanoLuc to generate a bright luminescent signal in the presence of furimazine (Dixon et al., 2016). We tagged the HiBiT sequence to tau, allowing for sensitive detection of secreted tau upon addition of LgBiT into the medium. Our results show that both native and pseudohyperphosphorylated tau are released from cultured hippocampal neurons. Pharmacological stimulation of neuronal activity led to a modest increase in wild-type and mutant tau release, while silencing activity had no effect. We further show that HSPGs contribute to the release of both native and pseudohyperphosphorylated tau. Altogether this suggests that native and mutant tau are released via overlapping mechanisms; however, the release of pseudohyperphosphorylated tau was more efficient than that of wild-type tau under all conditions.

## Results

### Development of a HiBiT-tau reporter system for the detection of tau secretion *in vitro*

Tau is a cytosolic microtubule-binding protein that is not targeted for conventional secretion via the endoplasmic reticulum–Golgi route. It is, however, well-established that both native and pathogenic tau are secreted (Yamada et al., 2011; Pooler et al., 2013; Wu et al., 2016). It remains unclear to what degree underlying unconventional secretion mechanisms that allow its release into the extracellular space overlap between native and pathogenic tau. We thus set out to design a sensitive assay that reports on tau secretion independent of its re-uptake and propagation of misfolding in subsequent cells. We chose a luminescence-based reporter system as it is based on an enzymatic reaction providing amplification and thus high sensitivity, combined with a large dynamic range.

**FIGURE 1:**
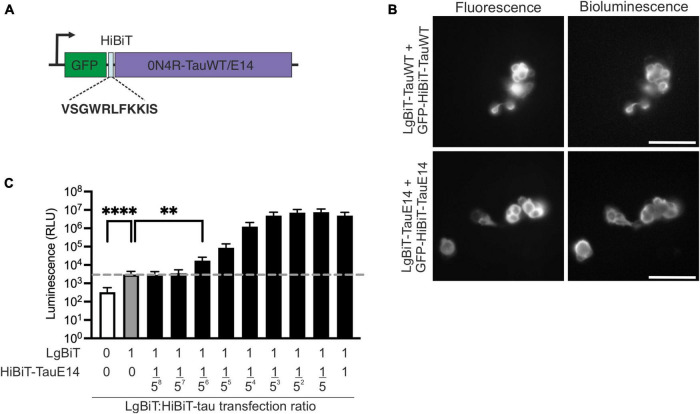
Generation of the HiBiT-tau biosensor. **(A)** Schematic diagram of the HiBiT-tau construct indicating the HiBiT amino acid sequence. **(B)** Fluorescence and bioluminescence imaging of HEK293 cells co-transfected with GFP-HiBiT-tau^WT^ or GFP-HiBiT-tau^E 14^ and LgBiT- tau^WT^ and LgBiT- tau^E 14^. Scale bar, 100 μm. **(C)** HEK293 cells were co-transfected with serial dilutions of GFP-HiBiT-tau^E 14^ and constant amount of LgBiT. Dotted line, signal generated by LgBiT in absence of HiBiT (gray bar, “1:0”) above background (white bar); *p* < 0.0001. GFP-HiBiT-tau^E 14^ is first detectable above background at a dilution of 1:15,625 (“1:1/5^6^”); *p* = 0.01, one-way ANOVA followed by Dunnet’s comparison test, (*n* = 3). **p < 0.01; ****p < 0.0001.

To investigate the secretion of native and disease-associated tau, we modified GFP-tau 0N4R wild-type (WT) and E14, containing 14 serine/threonine sites mutated into glutamate (Hoover et al., 2010), by inserting the HiBiT sequence between GFP and tau (Figure 1A) and cloning the chimeric open reading frame into a lentiviral backbone. Bioluminescence and fluorescence imaging following co-expression of these new constructs with LgBiT-Tau in HEK293 cells revealed successful integration of the HiBiT sequence into the constructs, as well as accessibility of the HiBiT tag for complementation (Figure 1B). In order to assess tau secretion, an excess of LgBiT will need to complement with limiting amounts of GFP-HiBiT-tau and generate a signal above the background generated by the presence of LgBiT alone. To gain a first estimate of sensitivity of this system, we co-transfected HEK293 cells with LgBiT and decreasing amounts of GFP-HiBiT-tau^E14^. We observed that LgBiT by itself (Figure 1C, gray bar) produced a signal above background (Figure 1C, white bar; indicated by dashed line, *p* < 0.0001). Addition of GFP-HiBiT-tau^E14^ led to a concentration-dependent increase in bioluminescent signal above background (Figure 1C, black bars). Importantly, already very low levels of GFP-HiBiT-tau^E14^ co-transfection allowed a robust detection of luminescence signal above that generated by LgBiT alone, beginning at 1 LgBiT: 1/5^6^ HiBiT-tau^E14^ (*p* = 0.01, Figure 1C), suggesting that the assay has the sensitivity needed to report on low levels of tau secretion.

**FIGURE 2:**
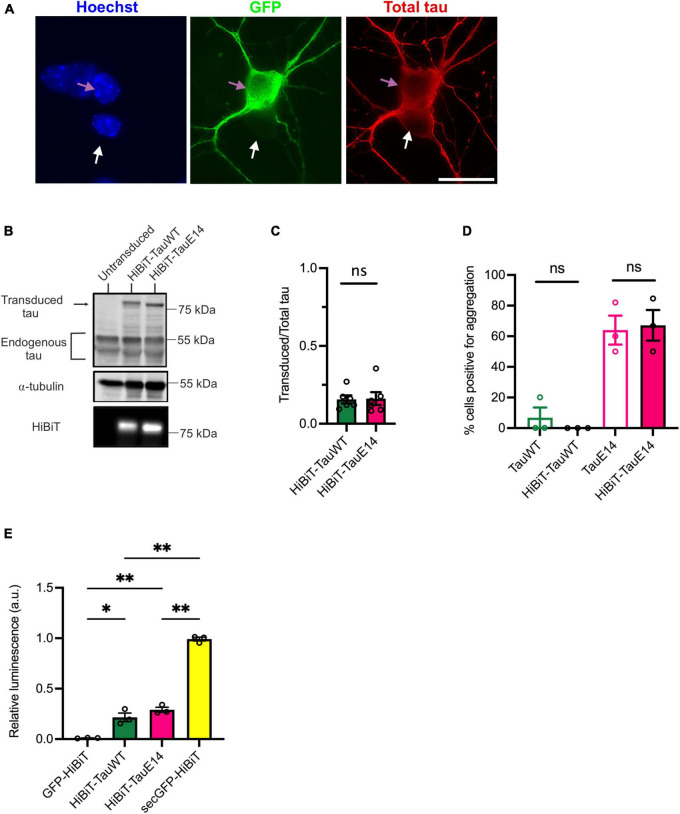
Characterization of the HiBiT-tau biosensor in primary neurons. **(A)** Representative images of DIV14 hippocampal neurons transduced at DIV1 with lentivirus to expresses GFP-HiBiT-tau^WT^. Cultures were fixed at DIV14 and stained with anti-tau antibody (red) and DAPI (blue). Pink arrow highlights a transduced, white arrow an untransduced neuron. Scale bar, 20 μm. **(B)** Western blot showing presence of transduced tau and endogenous tau in cell lysates of cultured DIV14 hippocampal neurons. The presence of HiBiT was detected through bioluminescence imaging (lower panel). **(C)** Densitometry of the immunoblot showing relative expression of transduced tau over total (endogenous and transduced) tau for GFP-HiBiT-tau^WT^ and GFP-HiBiT-tau^E 14^ reveals no significant difference in relative expression (*p* = 0.31; two-tailed paired *t*-test, *n* = 6). **(D)** Analysis of tau aggregation within the axon in absence versus presence of HiBiT (GFP-tau^WT^ vs. GFP-HiBiT-tau^WT^, *p* = 0.99; GFP-tau^E 14^ vs. GFP-HiBiT-tau^E 14^, *p* = 0.99; one-way ANOVA, followed by Holm-Sidak’s multiple comparisons, *n* = 3). **(E)** GFP-HiBiT, GFP-HiBiT-tau^WT^, GFP-HiBiT-tau^E 14^ and secGFP-HiBiT secretion was assessed in conditioned media collected from DIV13-15 cultured hippocampal neurons. ns, not significant; *p < 0.05; **p < 0.01; GH, GFP-HiBiT.

To validate the new HiBiT-tau constructs in neurons, DIV1 primary hippocampal neurons were transduced and expression was assessed at DIV14 by immunostaining and western blot analysis (Figures 2A–C). This revealed that the transduced GFP-HiBiT-tau^WT^ and GFP-HiBiT-tau^E14^ were present at equivalent levels and contributed a minor fraction to the total cellular tau pool (16%, Figure 2C). Bioluminescent imaging of the blot membrane following incubation with LgBiT further confirmed successful integration of the HiBiT sequence (Figure 2B, bottom panel). To ensure that the fusion of the HiBiT sequence does not alter tau misfolding, we compared the fluorescence distribution of GFP-HiBiT-tau^WT^, GFP-tau^WT^, GFP-HiBiT-tau^E14^ and GFP-tau^E14^ at the distal axon as described previously (Hallinan et al., 2019). We observed spontaneous misfolding of tau^E14^, while tau^WT^ retained a smooth distribution. Further, we detected no difference between the tau constructs containing or missing the HiBiT sequence (Figure 2D), confirming that the new chimeric tau proteins display all expected characteristics. Finally, to enable comparison of tau secretion under different conditions, it must be detectable above background but below saturation within a dynamic range. To assess if this is the case, we transduced primary hippocampal neurons at DIV1 with GFP-HiBiT, GFP-HiBiT-tau^WT^, GFP-HiBiT-tau^E14^ or secGFP-HiBiT that is targeted into the secretory pathway. Conditioned medium was analyzed upon LgBiT addition. This demonstrated that both GFP-HiBiT-tau^WT^ and GFP-HiBiT-tau^E14^ are detectable above background but at levels well below that of a protein targeted to the conventional secretory pathway (Figure 2E), suggesting that the assay is valid for monitoring and comparing tau secretion.

### Tau is secreted in vesicular and free form

**FIGURE 3:**
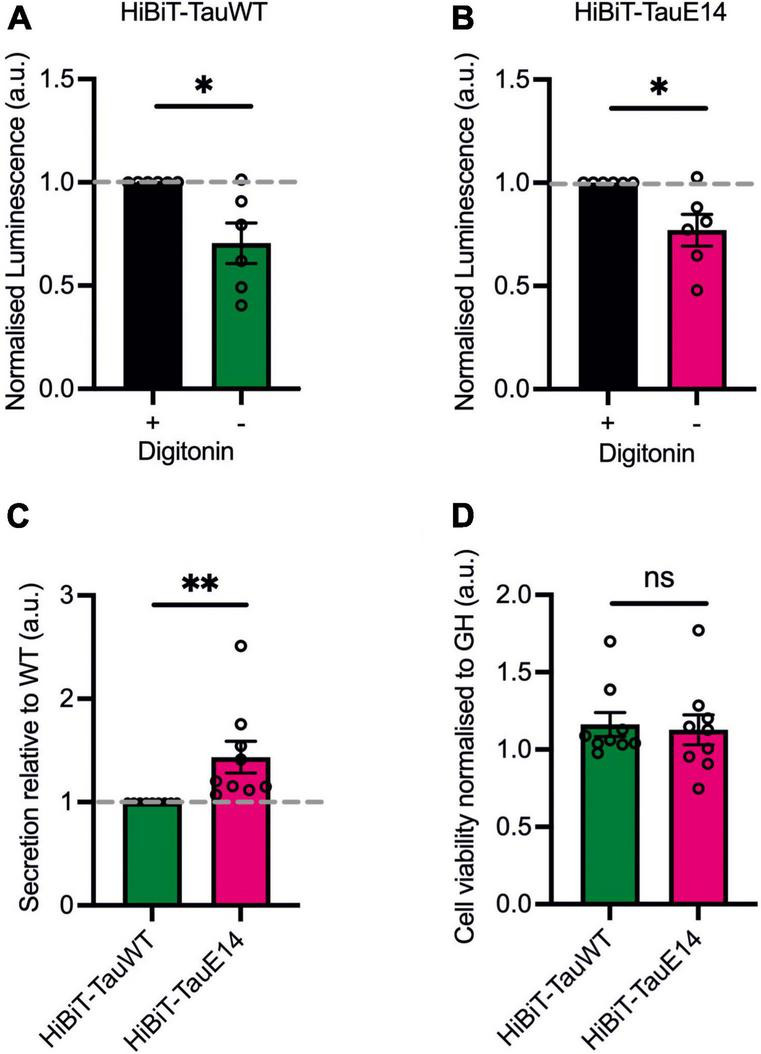
The majority of tau is secreted as a free protein. **(A,B)** GFP-HiBiT-tau^WT^ **(A)** and GFP-HiBiT-tau^E 14^ **(B)** secretion was assessed in conditioned media collected from DIV13-15 cultured hippocampal neurons in presence or absence of digitonin. There is a significant difference between non-lytic and lytic luminescence signal in GFP-HiBiT-tau^WT^ (*p* = 0.015) and GFP-HiBiT-tau^E 14^-conditioned media (*p* = 0.016; paired *t*-test, *n* = 6). **(C)** Differential secretion of free tau between GFP-HiBiT-tau^WT^ and GFP-HiBiT-tau^E 14^ (*p* = 0.008, two-tailed paired *t*-test, *n* = 9). **(D)** Calcein blue AM was used to assess cell viability between GFP-HiBiT-tau^WT^ and GFP-HiBiT-tau^E 14^-expressing cultures. Fluorescence signals were normalized to GFP-HiBiT. No significant difference in cell viability (*p* > 0.999, paired *t*-test, *n* = 9) was observed. ns, not significant; *p < 0.05; **p < 0.01.

First, to assess if GFP-HiBiT-tau^WT^ and GFP-HiBiT-tau^E14^ are secreted within a vesicular structure or as a free protein, DIV1 murine hippocampal cultures were transduced with the respective lentivirus and secretion was analyzed at DIV13-15. Conditioned medium was collected from the transduced cultured hippocampal neurons and LgBiT was added to the medium in the absence (“non-lytic”) or presence (“lytic”) of the non-ionic detergent, digitonin, to differentiate the HiBiT-tau released as a free protein and the total amount of released tau, both in vesicles and free, in the extracellular medium. The addition of digitonin does not alter bioluminescent read-out (Supplementary Figure 1). This revealed a clear increase in detected tau in the presence of digitonin, suggesting that a proportion of tau is secreted within a membrane structure (Figures 3A, B; GFP-HiBiT-tau^WT^, *p* = 0.015 and GFP-HiBiT-tau^E14^, *p* = 0.016, paired *t*-test, *n* = 6). However, the membrane-free tau made up the predominant pool of detected tau for both GFP-HiBiT-tau^WT^ (71 ± 10%) and GFP-HiBiT-tau^E14^ (77 ± 8%) (Figures 3A, B). We thus decided to focus on the secretion of free tau protein in subsequent experiments.

### Tau^E14^ is preferentially secreted over tau^WT^

To compare the secretion between GFP-HiBiT-tau^E14^ and GFP-HiBiT-tau^WT^, the level of GFP-HiBiT-tau detected in the conditioned medium of cultured hippocampal neurons expressing GFP-HiBiT-tau^WT^ or GFP-HiBiT-tau^E14^ was analyzed (Figure 3C). GFP-HiBiT was expressed in parallel wells as a cytosolic protein, and signal detected in the medium collected from GFP-HiBiT expressing neurons is a consequence of the combination of unspecific background and disintegrating cells within the culture. This signal was thus subtracted from the NanoLuc signal generated by condition media collected from GFP-HiBiT-tau^E14^ and GFP-HiBiT-tau^WT^ expressing cultures (Figure 3C). To account for variation in tau protein expression, immunostaining and western blotting analysis were used and revealed no significant differences between the conditions (Figures 2A–C). This revealed modestly increased levels of pseudohyperphosphorylated tau relative to wild-type tau (Figure 3C; 43.5 ± 15.5%, *p* = 0.004, 2-tailed *t*-test, *n* = 9). To ensure that this is not the consequence of cytotoxicity of the mutant and increased cell disintegration we measured the density of viable cells using calcein blue AM. This showed that mutant tau did not affect cell viability (Figure 3D), as observed previously (Hallinan et al., 2019).

**FIGURE 4:**
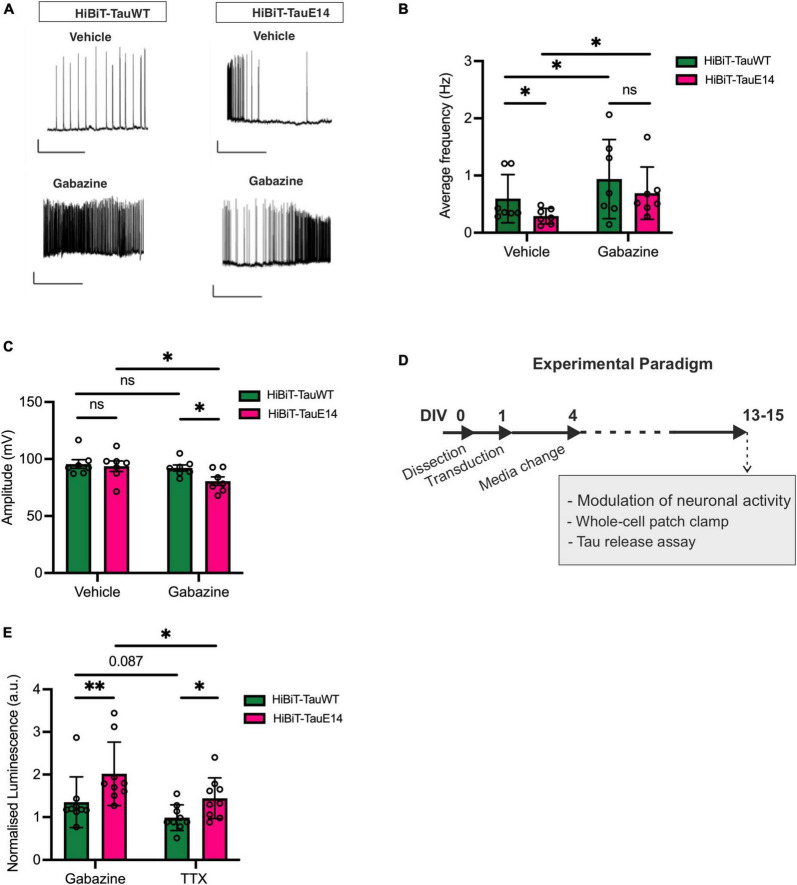
Activity-dependent secretion of GFP-HiBiT-tau^WT^ and GFP-HiBiT-tau^E 14^. **(A–C)** Whole-cell patch clamp recordings from transduced hippocampal neurons at DIV13-15. **(A)** Representative traces. Scale bar = 50 ms, 20 mV. **(B)** Average firing frequency (GFP-HiBiT-tau^WT^ vs. GFP-HiBiT-tau^E 14^ in vehicle: *p* = 0.018, gabazine: *p* = 0.037; vehicle vs. gabazine for GFP-HiBiT-tau^WT^: *p* = 0.012, GFP-HiBiT-tau^E 14^: *p* = 0.006; two-way ANOVA followed by Fisher’s LSD test, *n* = 7) and **(C)** action potential amplitude (two-way ANOVA followed by Sidak’s test, *n* = 7). **(D)** Schematic diagram of the experimental paradigm. **(E)** Activity-dependent secretion of tau from neuronal cultures expressing GFP-HiBiT-tau^WT^ or GFP-HiBiT-tau^E 14^ relative to GFP-HiBiT-tau^WT^ vehicle treatment (TTX vs. gabazine for GFP-HiBiT-tau^WT^: *p* = 0.087, GFP-HiBiT-tau^E 14^: *p* = 0.018; GFP-HiBiT-tau^WT^ vs. GFP-HiBiT-tau^E 14^ in TTX: *p* = 0.045, gabazine: *p* = 0.008; two-way ANOVA followed by Fisher’s LSD test; *n* = 7). ns, not significant; *p < 0.05; **p < 0.01.

Activity-dependent secretion of tau has been documented (Pooler et al., 2013; Yamada et al., 2014; Wu et al., 2016). Since mutant tau dampens neuronal activity *in vivo* (Busche et al., 2019), we next investigated the baseline spontaneous activity in cultures transduced with GFP-HiBiT-tau^WT^ or GFP-HiBiT-tau^E14^. As described previously (Hallinan et al., 2019), both GFP-HiBiT-tau^WT^ or GFP-HiBiT-tau^E14^ expressing cultures showed basal activity (Figures 4A, B) with no difference in intrinsic membrane properties including the resting membrane potential, input resistance and rheobase (data not shown). Analysis revealed a reduced action potential frequency in cultures expressing GFP-HiBiT-tau^E14^ (*p* = 0.018, two-way ANOVA, followed by Fisher’s LSD, Figures 4A, B) but no effect on the amplitude (Figure 3C). To alter baseline activity, cells were incubated with either 500 nM tetrodotoxin (TTX), which abolished action potentials, or 3 μM gabazine, a γ-aminobutyric acid (GABA) A receptor inhibitor, to increase excitability (Figures 4A–D). Gabazine treatment increased the firing frequency in both GFP-HiBiT-tau^WT^ or GFP-HiBiT-tau^E14^ expressing cultures (Figures 4A, B) and led to a decrease in amplitude in the presence of GFP-HiBiT-tau^E14^ (*p* = 0.019, two-way ANOVA followed by Sidak’s test, Figure 4C). When normalized to vehicle-treated GFP-HiBiT-tau^WT^ expressing cultures, GFP-HiBiT-tau^WT^ secretion was trending higher in the presence of gabazine compared to TTX (*p* = 0.087), and GFP-HiBiT-tau^E14^ secretion was increased with elevated activity (*p* = 0.018, two-way ANOVA, followed by Fisher’s LSD, Figure 4E). The direct comparison of GFP-HiBiT-tau^WT^ and GFP-HiBiT-tau^E14^ showed that pseudohyperphosphorylated tau was secreted at higher levels than wild-type tau both in the absence of action potentials and following gabazine treatment (Figure 4E), despite the comparatively lower action potential frequency and amplitude (Figures 4B, C). No difference in cell viability was detected (data not shown). Interestingly, when compared to their own vehicle-treated conditions, neither GFP-HiBiT-tau^WT^ nor GFP-HiBiT-tau^E14^ showed a reduction in secretion in the presence of TTX (Supplementary Figure 2). Combined with the modest effect of gabazine treatment, this suggests that activity-independent secretion of tau primarily contributes to its release.

### Heparan sulfate proteoglycans play a major role in the secretion of both pseudohyperphosphorylated and wild-type tau

**FIGURE 5:**
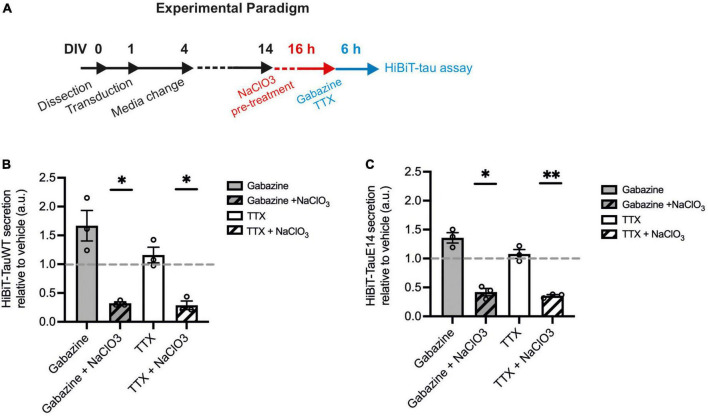
HSPG inhibition leads to reduced tau secretion. **(A)** Schematic diagram of the experimental paradigm. **(B)** Luminescence measured in cultures expressing GFP-HiBiT-tau^WT^ following indicated treatments, relative to vehicle control (gabazine vs. gabazine + NaClO_3_, *p* = 0.030; TTX vs. TTX + NaClO_3_, *p* = 0.038; gabazine + NaClO_3_ vs. TTX + NaClO_3_, *p* = 0.55, one-way ANOVA followed by Fisher’s LSD test, *n* = 3). **(C)** Luminescence measured in cultures expressing GFP-HiBiT-tau^E 14^ following indicated treatments, relative to vehicle control (gabazine vs. gabazine + NaClO_3_, *p* = 0.025; TTX vs. TTX + NaClO_3_, *p* = 0.0076; gabazine + NaClO_3_ vs. TTX + NaClO_3_, *p* = 0.50, one-way ANOVA followed by Fisher’s LSD test, *n* = 3). *p < 0.05; **p < 0.01.

There is growing evidence to suggest that tau can directly translocate across the plasma membrane in primary neurons and immortal cell lines (Katsinelos et al., 2018; Merezhko et al., 2018). How this mechanism interacts with activity-dependent secretion remains unclear. To investigate this interaction, HSPG biosynthesis was blocked using sodium chlorate (NaClO_3_) in cells expressing GFP-HiBiT-tau^WT^ and GFP-HiBiT-tau^E14^ (Figure 5A). Sodium chlorate selectively affects the sulfation patterns of heparan sulfates (Safaiyan et al., 1999) without affecting cell viability (Supplementary Figure 3). As observed before, TTX treatment did not lower the secretion of either tau variant, while gabazine led to a modest increase in release relative to vehicle treatment (Figures 5B, C). Pre-treatment with NaClO_3_ significantly lowered secretion of GFP-HiBiT-tau^WT^ both in the presence of gabazine (to 32.1 ± 2.7% of vehicle; *p* = 0.0016) and TTX (to 28.7 ± 7.5% of vehicle, *p* = 0.011, one-way ANOVA followed by Fisher’s LSD test, Figure 5B). Moreover, following NaClO_3_ pre-treatment no difference in GFP-HiBiT-tau^WT^ release was detected between silenced cultures compared to those with enhanced activity, suggesting that the activity-dependent component in tau release is entirely HSPG-dependent (Figure 5B). As previously reported, GFP-HiBiT-tau^E14^ secretion was elevated compared to GFP-HiBiT-tau^WT^ (data not shown). As observed for GFP-HiBiT-tau^WT^, GFP-HiBiT-tau^E14^ secretion was significantly reduced by pre-treatment with NaClO_3_ both in the presence of gabazine (to 41.9 ± 6.4% of vehicle-treated cultures, *p* = 0.012) or TTX (to 35.5 ± 2.0% of vehicle, *p* = 0.001, one-way ANOVA followed by Fisher’s LSD test, Figure 5C). Again, the remaining GFP-HiBiT-tau^E 14^ release following NaClO_3_ pre-treatment was not modulated by changing the activity of the culture (Figure 5C). There was no difference in culture viability between any of the treatment groups, as measured by calcein blue AM (data not shown). Altogether this suggests that GFP-HiBiT-tau^WT^ and GFP-HiBiT-tau^E 14^ employ multiple overlapping mechanisms of release; their secretion is stimulated by activity and in large parts dependent upon HSPGs.

## Discussion

Tau propagation across neuronal networks requires the release from an affected cell, internalization into an adjacent cell, translocation into the cytosol prior to interaction with native tau, and its templated misfolding. A range of biosensors exist to detect the templated misfolding (Holmes et al., 2014; Hallinan et al., 2019), including luminescence-based assays (Cecon et al., 2023). Here we developed a NanoBiT-tau biosensor assay that allows the sensitive detection of tau localization. The biosensor is unique in that it does not rely on the presence of misfolding and interaction in adjacent cells to indicate that tau release and re-uptake has taken place, thus allowing the cell-to-cell transmission of both native and pathogenic tau to be investigated at all its individual stages of release, re-uptake and interaction with native tau to template misfolding. In this study, we focused on tau release. Within the extracellular space the biosensor further allows free tau protein to be distinguished from vesicular tau. Here, we show that both physiological and pseudohyperphosphorylated tau are primarily released in free form but are also detectable in extracellular vesicles. While we focus on tau secretion in this study, the assay can readily be adapted to investigate the tau re-uptake process (Tuck et al., 2022); the expression of LgBiT in post-synaptic cells allows the re-uptake of both wild-type and mutant tau to be monitored as it enters the cytosol. This enables the detection of the early events that precede seeded aggregation. In comparison to fluorescence-based biosensors and biochemical approaches, the NanoBiT-tau reporter assay has a high sensitivity, excellent signal-to-noise ratio and is readily customizable to different assay formats (Hall et al., 2021).

We employed this assay to compare the release mechanisms of physiological and pseudohyperphosphorylated tau. We assessed the contribution of activity to the secretion of native and mutant tau. Our data suggest that secretion is largely independent of neuronal activity as application of TTX to silence action potentials did not result in a decrease of physiological or pathological tau release. This is in line with previous observations delivering TTX via microdialysis *in vivo* (Yamada et al., 2014). Conversely, increasing activity by blocking inhibition led to a modest increase of secretion of both forms of tau. Previous studies suggested a stronger effect of activity on tau release (Pooler et al., 2013; Wu et al., 2016; Ismael et al., 2021) which may be the result of several differences in experimental approach. First, the elevation of K^+^ as a proxy of activity leads to depolarization and Ca^2+^ influx, but not enhanced neuronal firing (Supplementary Figure 4). The non-physiological high Ca^2+^ influx under these conditions may drive stronger release, and frequently leads to subsequent cell death. However, a stronger effect on tau release has also been documented following optogenetic stimulation of neuronal activity, resulting in enrichment of tau in conditioned media assessed by western blotting. We propose that the direct read-out and large dynamic range of the bioluminescence-based assay render it more reflective of actual fold changes, which are not dissimilar to those observed by ELISA *in vivo* (Yamada et al., 2014). Propagation of tau pathology across neuronal networks has also been shown to be enhanced by activity (Wu et al., 2016). Here, activity may increase propagation not only at the step of tau secretion, but potentially also contribute to internalization into the connected neuron as well as the templated misfolding.

There are numerous studies showing that tau can bind to HSPGs (Holmes et al., 2013; Katsinelos et al., 2018; Merezhko et al., 2018; Rauch et al., 2018). HSPGs have been shown to play an important role in the spread of tau pathology in neuronal networks (Holmes et al., 2013; Rauch et al., 2018), and reducing HSPGs decreases tau secretion (Katsinelos et al., 2018; Rauch et al., 2018). Here, we show that NaClO_3_ treatment of the neuronal cultures to prevent HSPG biosynthesis completely abolished the effect of neuronal activity on tau secretion as well as diminishing the activity-independent release.

A previous study proposed that tau^E 14^ secretion is elevated because of its reduced affinity to microtubules and therefore increased availability to interact with the inner leaflet of the plasma membrane to be positioned for translocation (Katsinelos et al., 2018). In accordance with this suggestion, we see higher levels of secretion for the pseudohyperphosphorylated tau compared to wild-type. In summary, we developed a scalable biosensor assay that allows monitoring tau localization with high sensitivity and a large dynamic range, independent of its oligomerization. Using this assay, we showed that native and mutant tau employ multiple overlapping mechanisms of release from primary neurons: both are primarily secreted as a free protein, likely the result of direct translocation across the membrane. Both show a modest increase in release in response to increased neuronal activity and an almost complete block of secretion in response to sodium chlorate treatment.

## Materials and methods

### Plasmids

pRK5-GFP-tau^WT^ and pRK5-GFP-tau^E 14^ were gifted from Karen Ashe (Hoover et al., 2010; Addgene, plasmids #46904 and #46907); split NanoLuc plasmids were from Promega. pRK5-GFP-HiBiT-tau^WT^ and pRK5-GFP-HiBiT-tau^E 14^ were created by annealing forward oligonucleotide (5′-GATCCTCCGGCGGCGG CGGCTCCGTGTCCGGCTGGCGGCTGTTCAAGAAGATCTCC TCCGGCGGCGGCTCCA-3′) and reverse oligonucleotide (5′-GA TCTGGAGCCGCCGCCGGAGGAGATCTTCTTGAACAGCCGC CAGCCGGACACGGAGCCGCCGCCGCCGGAG-3′) encoding the HiBiT sequence, flanked by *Bam*HI N-terminus and BgllI C-terminus overhang. HiBiT was cloned into the pRK5 vector backbone using a *Bam*HI restriction site located at the N-terminus of GFP and C-terminus of tau. pRK5-LgBiT-tau^WT^ and pRK5-LgBiT-tau^E 14^ were generated by removing GFP from the pRK5-GFP-HiBiT-tau^WT^ and pRK5-GFP-HiBiT- tau^E 14^ plasmids using *Cla*I and *Bam*HI and was replaced with LgBiT isolated from pBiT1.1-N[TK/LgBiT] (Promega, UK, N2014) using forward primer (5′-ATCGATGCCACCATGGTCTTCACACTCGAAGAT-3′) and reverse primer (5′-GGATCCTCCACCGCTCGAGCCTCCACC-3′). pRK5-GFP-HiBiT was generated by removing the N-terminal tau of the pRK5-GFP-HiBiT-tau^E 14^ plasmid via site-directed mutagenesis using forward primer (5′-TGAGTCGAC CTGCAGAAG-3′) and reverse primer (5′-GCCGCCGG AGCTAATCTT-3′). To generate lentiviral expression plasmids, GFP-HiBiT-tau^WT^ and GFP-HiBiT-tau^E 14^ were amplified by PCR using forward primer 5′-ATCGATGGTCGCCACCATGGTGA-3′ and reverse primer 5′-TCTAGATCACAAACCCTGCTTGGCCA-3′ and were inserted between *Cla*I and *Xba*I restriction sites of pLV-EF1a-SV40 vector.

### Cell culture

All animal work was performed in accordance with the Animals Scientific Procedures Act 1986 set out by the UK Home Office. Primary cultures were prepared from C57BL/6 mouse hippocampus, taken from embryonic day (E)15-18 as previously described (Deinhardt et al., 2011). Cells were plated in neurobasal medium supplemented with 2% B27 Plus (Gibco, ThermoFisher Scientific, UK, A3582801) and 0.5 mM GlutaMAX (ThermoFisher Scientific, UK, 12348017) at a density of 133 cells/μl per well in a 96-well plate (ThermoFisher Scientific, 136101) and 150 cells/μl in a 48-well plate, pre-coated with 0.1 mg/ml poly-D-lysine (Sigma, UK, P0296-100MG). HEK293FT cells were grown in DMEM supplemented with 10% FBS.

### Transfection and transduction

Primary neuronal cells were transfected at day *in vitro* (DIV) 1 with DNA and Lipofectamine 2000 (Invitrogen, ThermoScientific, UK) at a 1:1 ratio as described previously (Deinhardt et al., 2011). At DIV14, transfected neuronal axons were assessed for the presence of aggregation as previously described (Hallinan et al., 2020). HEK293 cells were transfected 24 h after plating at a 1:3 DNA and Lipofectamine 2000 ratio. 24 h post-transfection, cells were used for western blotting and bioluminescence imaging. For assay sensitivity experiments, pBiT1.1-N[TK/LgBiT] (Promega) plasmid was co-transfected with pRK5-GFP-HiBiT- tau^E 14^.

Viruses were produced by transfecting HEK293FT cells with Gag Polymerase, VSV-G and pLV-EF1a-SV40 at a ratio of 4:2:1 using Lipofectamine 2000. After 48 h post-transfection, media was replaced with fresh DMEM with 10% FBS. Media containing virus was collected 48 h later and centrifuged at 600 rcf to pellet cell debris. Plated primary neurons were transduced at DIV1 by replacing 50% of media with lentiviral media, followed by a complete media change after 72 h. Assays were performed at DIV13-15. All viral batches were checked for even transduction efficiency and equivalent expression of GFP-HiBiT- tau^WT^ and GFP-HiBiT- tau^E 14^ using western blot.

### SDS PAGE, western blot and HiBiT blotting

HEK293 cells were lysed in sample buffer [62.5 mM Tris-HCl pH 6.8, 2% (v/v) SDS, 10% (v/v) glycerol, 5 mM dithiothreitol and 0.001% (v/v) bromophenol blue dye] and analyzed by western blot using primary antibodies for tau (1:1000; DAKO, Agilent, USA, A0024) and α-tubulin (1:1000, Sigma, B5-1-2). The membranes were scanned with Odyssey Infrared Imaging Scanner (LICOR, USA) and Image Studio Light Software (LICOR) was used to analyze the image. To detect HiBiT, membranes were incubated in Nano-Glo HiBiT Blotting Buffer (Promega, N2410) containing LgBiT followed by addition of furimazine, and imaged using a luminescence imager with a CCD camera.

### Immunocytochemistry

Cells were fixed in 4% paraformaldehyde for 10–15 min, quenched with 50 mM NH_4_Cl in TBS for 5 min and permeabilized in 0.1% Triton X-100 in TBS for 5 min at room temperature (RT). Cells were blocked with 10% normal goat serum and 2% bovine serum albumin in TBS at RT for 45 min, incubated with relevant antibodies (anti-tau, 1:1000, DAKO A0024; secondary antibodies, 1:10 000, Invitrogen), stained with Hoechst (ThermoFisher 33342) and mounted on using Mowiol488.

### Microscopy and image analysis

Images for axonal analysis were taken using a Nikon E800 upright fluorescence microscope, equipped with a Plan Fluor 60 × 1.40 NA oil immersion objective lens (Nikon Instruments Inc., Japan) and an Imaging OptiMOS sCMOS 1,920 × 1,080 pixel camera (Photometrics, USA). Aggregate analysis along axons was performed as previously described (Hallinan et al., 2020). Bioluminescence images were collected using DM-IRB inverted microscope stand (Leica Microsystems, Germany), equipped with a Plan Fluotar 40 × 0.50–1.00 NA oil objective (Leica Microsystems) and an Imaging Rolara Thunder EMCCD camera with a 512 × 512 pixel chip (Photometrics, USA). All image acquisition was achieved using Micro-manager software (University of California, San Francisco, USA), and images were processed and analyzed using ImageJ or Fiji (NIH, USA).

### HiBiT-tau assays

To assess the sensitivity of the bioluminescence assay, HEK293 cells were plated on white 96-well plates and transfected with plasmids encoding LgBiT and indicated dilutions of pRK5-GFP-HiBiT-tau^E 14^. To measure basal secretion, primary neurons on white 96-well plates were transduced at DIV1 with lentivirus for expression of GFP-HiBiT, GFP-HiBiT-tau^WT^ or GFP-HiBiT-tau^E 14^. At DIV4, a complete media change was performed and media was harvested at DIV13-15. All measurements were taken as triplicates, and positions on the 96-well plate were rotated between experiments to avoid bias due to positioning.

To assess neuronal activity, the media was exchanged for artificial cerebrospinal fluid (ACSF: 10 mM HEPES-NaOH pH 7.3–7.4, 126 mM NaCl, 3 mM KCl, 1.25 mM NaH_2_PO_4_, 2 mM MgSO_4_, 2 mM CaCl_2_, 26 mM NaHCO_3_, and 10 mM glucose) with or without addition of 500 nM tetrodotoxin (TTX), or no MgSO_4_ and addition of 3 μM gabazine for indicated incubation times. To assess the contribution of heparan sulfate proteoglycans (HSPGs) to tau release, cells were pre-treated with 50 mM NaClO_3_ for 16 h prior to the treatments described above. An osmolarity of ∼300 mOsm/L was maintained for all solutions.

For bioluminescence detection, Nano-Glo HiBiT Extracellular Detection System (Promega, N2420) was added to measure the HiBiT-tau content. After 2 min incubation, the plate was read by the ClarioSTAR plate reader at RT with 5 s integration (emission 460 nm). The GFP-HiBiT condition was used for background subtraction across all conditions to account for release due to cell death within the cultures prior to normalization of values as indicated in each figure. To quantify the amount of free and vesicular tau in the media, the incubation with the Nano-Glo detection system was performed in the absence or presence of 50 μg/ml digitonin to permeabilize vesicular membranes. Prior to HiBiT-tau assay read-out, conditioned media was incubated with digitonin for 15 min.

### Cell viability

Following bioluminescence acquisition, 10 μM Calcein Blue AM (Invitrogen, C1429) was added to the cells, incubated for 20 min at 37°C and fluorescence intensity quantified using the ClarioSTAR plate reader (BMG Labtech, Germany) (excitation 360 nm; emission 449 nm, adjusted for background fluorescence). We did not detect variability in the viability measures between conditions within a plate, which would have served as exclusion criterion.

### Electrophysiology

Cells were cultured on coverslips and transduced with lentivirus as described above. For patch clamp recordings, cells were perfused at a rate of 1–2 ml/min with oxygenated (95% O2, 5% CO2) ACSF containing, in mM, 126 sodium chloride, 2 CaCl_2_, 10 glucose, 2 MgSO_4_, 3 KCl and 26.4 sodium carbonate. Patch clamp pipettes (5–7 MΩ) were pulled using thick-walled borosilicate glass tubing (World Precision Instruments) and filled with intracellular solution (in mM): 110 K-gluconate, 10 KCl, 10 Na-phosphocreatine, 10 HEPES, 4 ATP-Mg, 0.3 GTP, pH 7.25 adjusted with KOH; osmolarity at ∼280 mOsm/L. Recordings were performed at 25°C. Data were filtered at 5 kHz and acquired at 20 kHz with an Axopatch 200B amplifier (Molecular Devices) using MATLAB (Mathworks) and custom software (MatDAQ, Hugh P.C. Robinson, University of Cambridge 1995–2013). Intrinsic resting membrane potentials were measured and current was injected to maintain membrane potential of −70 mV, unless otherwise stated. Liquid junction potential of −13 mV was measured directly and corrected for. In current-clamp mode, increasing current injection steps of 25 pA up to 500 pA were used to test excitability. MATLAB was used in all data analysis.

### Data analysis

Axonal aggregation analysis were done previously described (Hallinan et al., 2019) and whole-cell patch clamp data were analyzed using custom MATLAB code. Biological repeats (*n*) were defined as the average of independent culture preparations from separate litters. In all HiBiT-tau assays and whole-cell patch clamp experiments, individual points represent the average of 2–3 technical replicates and 3–8 recorded cells from one biological repeat, respectively. Data from all HiBiT-tau assays were background subtracted using GFP-HiBiT signal. To account for differences in total tau protein expression all viral batches were tested using fluorescent imaging for transduction counts and western blotting to assess tau expression. To exclude variation in cell number, calcein blue AM fluorescence analysis was performed for each assessed well.

GraphPad Prism 6 was used (Ver 6.00m Graph Pad Software Inc., USA) to generate graphs and statistical analysis as described in the figure legends. Statistical significance was evaluated with paired *t*-test, one- or two-way ANOVA followed by Fisher’s LSD, Holm-Sidak’s or Dunnet’s test as indicated in the relevant figure legends. *T*-tests were two-tailed unless otherwise stated. Findings were considered significant as follows: *p < 0.05, ^**^p < 0.01, ^****^p < 0.0001. All error bars presented in graphs and range of values in the text are shown as standard error of mean (SEM).

## Data availability statement

The raw data supporting the conclusions of this article will be made available by the authors, without undue reservation.

## Ethics statement

All animal work was performed in accordance with the Animals Scientific Procedures Act 1986 set out by the UK Home Office and reviewed and approved by the University of Southampton Ethics Committee.

## Author contributions

DL, CM, MC, and KD designed the tau/split luciferase constructs. DL and CM generated and tested the constructs. ND designed the lentiviral generation. DL and KD designed the secretion assay and conditions, analyzed the data, and wrote the manuscript. DL performed the secretion assays. DL, HW, MV-C, and KD designed the electrophysiology experiments. DL and HW performed the electrophysiology experiments. DL, HW, and MV-C analyzed the electrophysiology data. All authors contributed to the article and approved the submitted version.
