# Supplementary material for: A luminescence-based reporter to study tau secretion reveals overlapping mechanisms for the release of healthy and pathological tau
Source: Front Neurosci. 2023 Jun 5;17:1196007. doi: 10.3389/fnins.2023.1196007 (PMC10277490; doi:10.3389/fnins.2023.1196007)
Supplement: Supplementary file 1 [file Image_1.pdf]

Supplementary material:

A luminescence-based reporter to study tau secretion reveals overlapping mechanisms for the release of healthy and pathological tau

Authors

Dianne Marquez Lopez[1], Connor J Maltby[1,2], Hannah Warming[1,3], Nullin Divecha[1], Mariana Vargas-Caballero[1], Mark J Coldwell[1,4], Katrin Deinhardt[1*]

Affiliations

[1]School of Biological Sciences, University of Southampton, UK

[2]current affiliation: Department of Neurology, University of Michigan, Ann Arbor, MI, USA

[3]current affiliation: Department of Physiology, Anatomy and Genetics, University of Oxford, Oxford, UK

[4]current affiliation: Promega UK Ltd, University of Southampton Science Park, UK

***E-mail:** K.Deinhardt@soton.ac.uk

Keywords

Tauopathy, tau secretion, split luciferase reporter, neuronal activity

Supplemental Figure 1

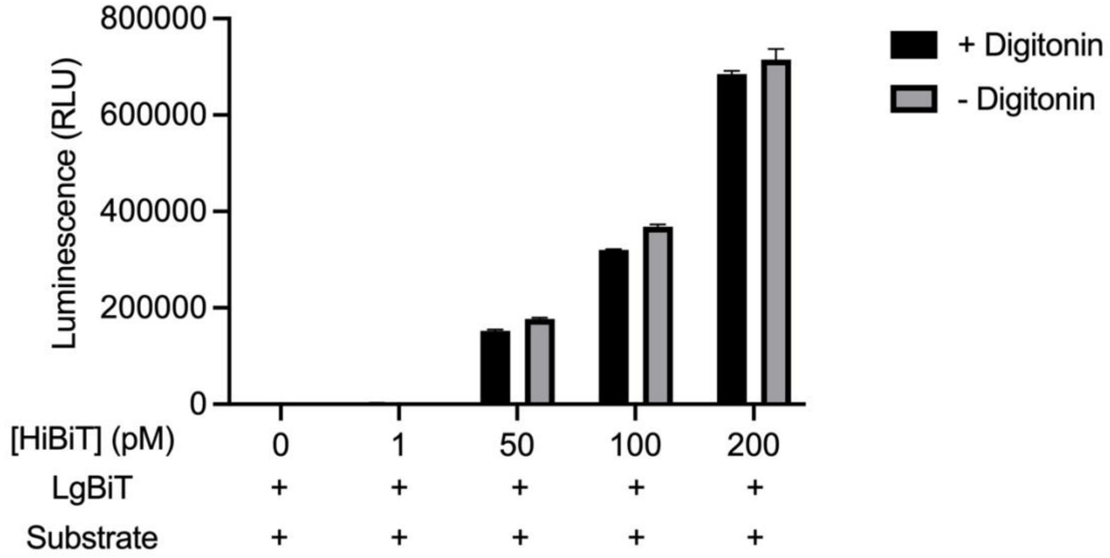

Supplemental figure 1. Comparable luminescence signals are generated by NanoBiT reporters in the presence and absence of digitonin. Increasing amounts of HiBiT peptide were mixed with a constant surplus of LgBiT protein prior to luminescence reading. Application of 50 µg/ml digitonin in the NanoBiT reporters does not alter the luminescence intensity of NanoLuc across a dynamic range of detection.

Supplemental Figure 2

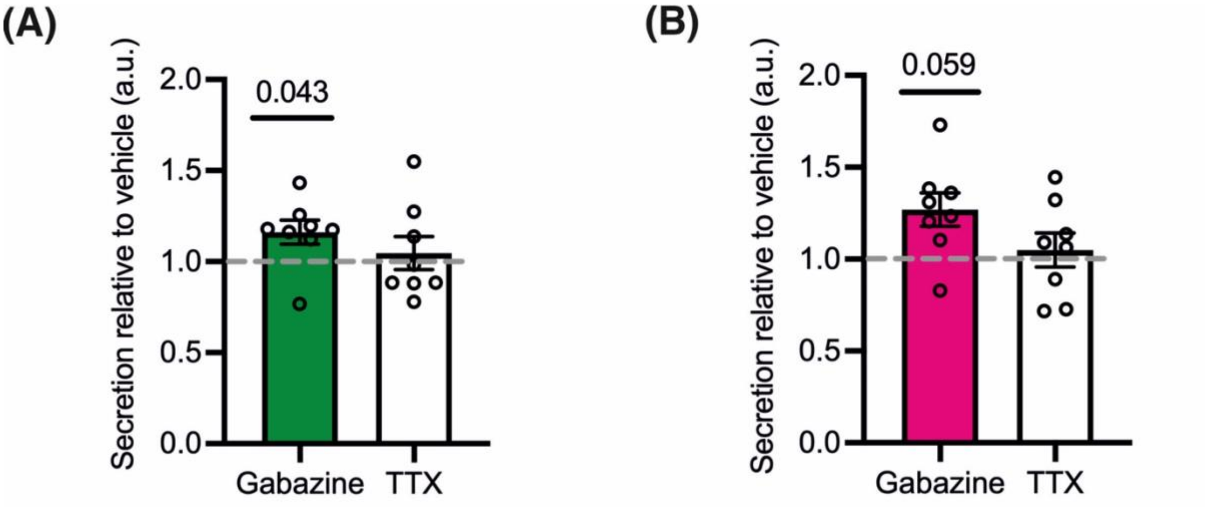

Supplemental figure 2. Inhibition of action potentials does not reduce tau secretion. Secretion of tau was measured as described for Figure 4E and normalised to its respective vehicle control. This shows that TTX has no effect on the release of wild-type (A) or pseudohyperphosphorylated (B) tau.

Supplemental Figure 3

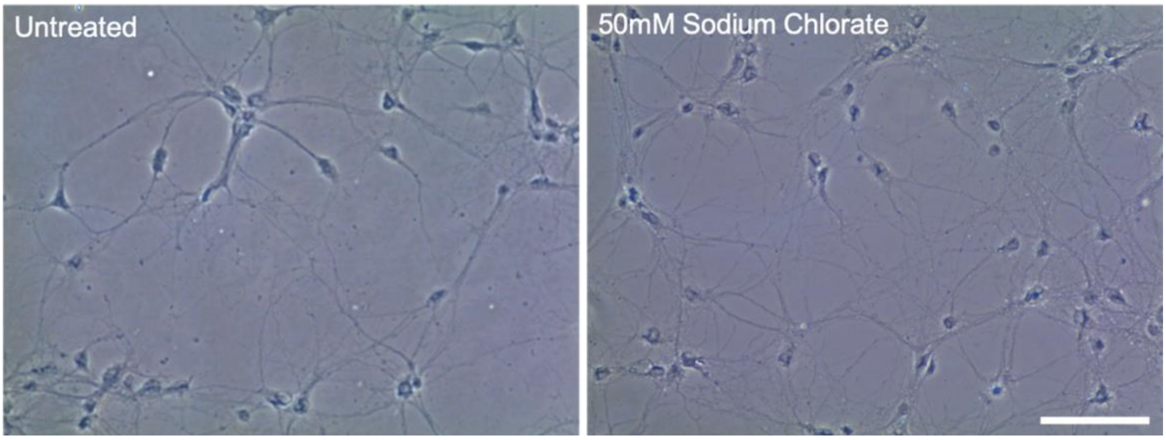

Supplemental figure 3: Application of sodium chlorate does not alter morphology of neurons. Brightfield microscope images of cultured hippocampal neurons pre-treated with or without 50 mM sodium chlorate for 16h. Scale bar = 50 µm.

Supplemental Figure 4

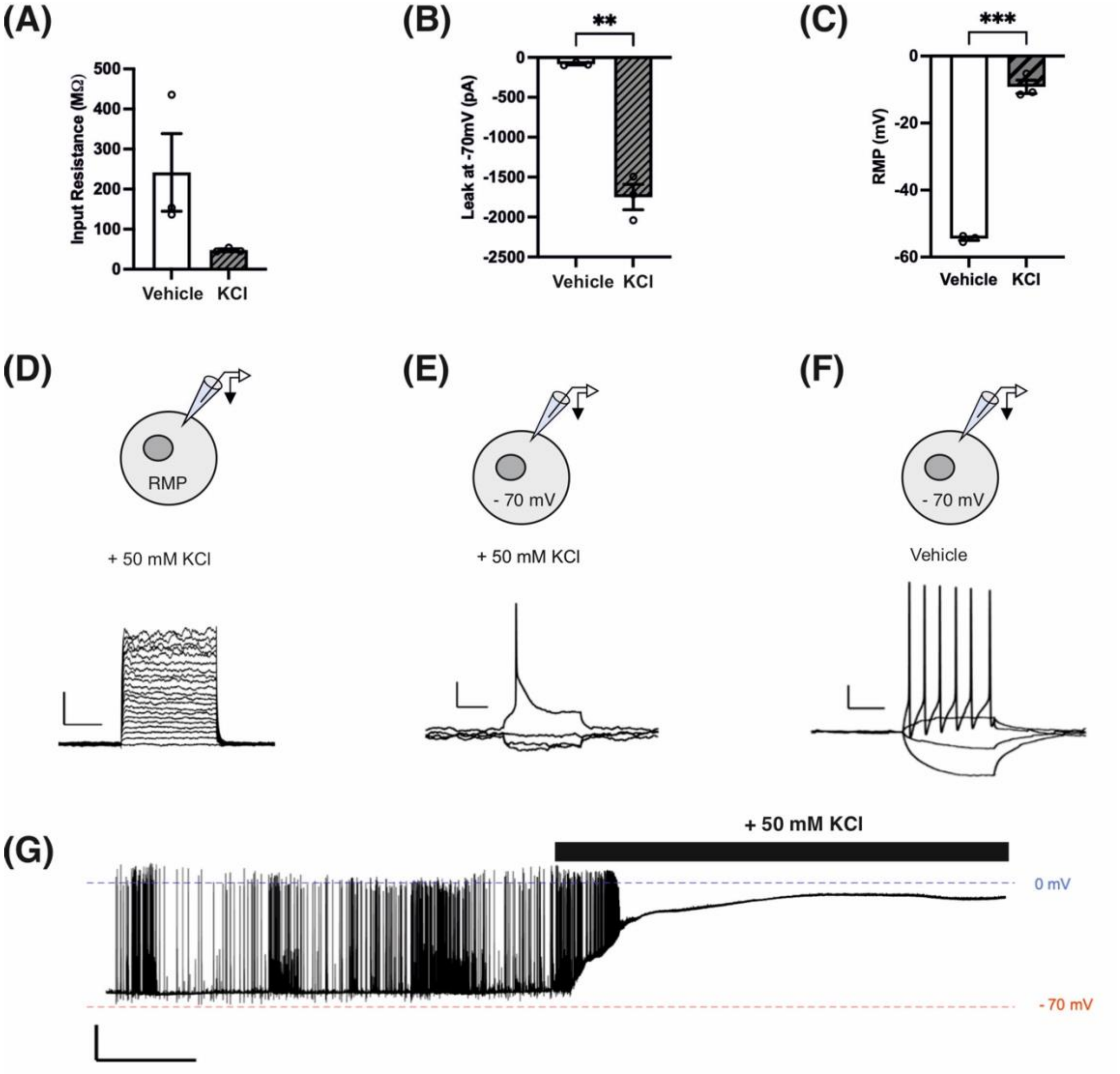

Supplemental figure 4. Elevating K^+ precludes action potential firing. Raising extracellular K^+ to 50 mM leads to (A) a decreased input resistance (p=0.086, paired t-test, n=3), (B) increased leak (p=0.0043, paired t-test, n=3) and (C) increased resting membrane potential (RMP; p=0.0006, paired t-test, n=3). (D) Under these conditions, no action potential could be evoked. (E) Upon correcting the membrane potential to -70 mV, a single action potential can be evoked, in contrast to (F) vehicle treated cells that show multiple consecutive action potentials. Scale bar = 50 ms, 20 mV. (G) Recording showing the depolarisation in response to elevated extracellular K^+. Scale bar = 100 s, 20 mV. **, p<0.01; ***, p<0.001
